# Transcriptional and Translational Inhibitors Block SOS Response and Shiga Toxin Expression in Enterohemorrhagic *Escherichia coli*

**DOI:** 10.1038/s41598-019-55332-2

**Published:** 2019-12-11

**Authors:** Michael Berger, Iqbal Aijaz, Petya Berger, Ulrich Dobrindt, Gerald Koudelka

**Affiliations:** 1 Institute of Hygiene, University of Münster, Münster, Germany; 2 Department of Biological Sciences, University at Buffalo, Buffalo, USA

**Keywords:** Transcription, Antibiotics

## Abstract

Shiga toxins (Stx) induce the symptoms of the life-threatening hemolytic uremic syndrome (HUS) and are the main virulence factors of enterohemorrhagic *Escherichia coli* (EHEC). The bacterial SOS response is the essential signal for high level production and release of Stx1/2. To assess the potential effectiveness of different antibiotics in blocking SOS response and Stx1/2 production, we constructed a reporter gene based test system that allows for the time-resolved, simultaneous read-out of the SOS response (*recA*P-*cfp*) and Stx1 production (*stx1::yfp*) in EHEC O157:H7 EDL933. We find that cells exposed to inhibitory or subinhibitory concentrations of ciprofloxacin did induce the SOS response, but not when the cells were exposed to rifaximine, azithromycin, tetracycline, gentamicin or ampicillin. Cell lysis and the peak in Stx1 production were substantially delayed with respect to the peak of the SOS response. We used this feature to show that adding transcriptional or translational inhibitors can block Stx1 production even after the SOS response is fully induced. RT-qPCR based tests with other clinically relevant EHEC isolates showed similar results for both Stx1 and Stx2. These observations suggest that transcriptional and translational inhibitors may be of value in treating EHEC infections.

## Introduction

Enterohemorrhagic *Escherichia coli* (EHEC) are foodborne pathogens. They are a significant cause of acute diarrheal illness with an estimated 2.8 million infections worldwide. EHEC pathogenesis is marked by the production and release of its essential virulence factor Shiga toxin (Stx)^1^. Human intoxication with Stx can induce the life-threatening hemolytic uremic syndrome (HUS). Due to its ability to cause HUS, EHEC are also the number one cause of renal failure in children in the United States^2^.

All EHEC strains harbor one or more temperate lambdoid Stx-encoding prophage. These prophage can encode either of two different Stx types: Stx1 and Stx2^3^. When present as a prophage, the genome of these phage lie essentially dormant within the host chromosome and activation of the lytic growth cycle is a rare event. When the DNA of the host cell is damaged, RecA polymerizes and forms a nucleoprotein complex that stimulates the autocatalytic cleavage of LexA, the repressor of *recA* and thereby induces its own expression. Similar to its effect on the CI of phage λ, polymerized RecA stimulates the autocatalytic cleavage of the repressor protein of the Stx-encoding prophage, which is crucial for the induction of the lytic growth cycle of the phage. Autocleavage of the repressor protein results in the activation of early and then the late phage genes, including *stx1/2*. During late lytic growth, the phage genome excises from the host chromosome, is packed into viral particles and these released from the cell upon lysis. The *stx2* genes are under the control of promoters that are active exclusively during the later stages of lytic growth. Thus Stx2 is only produced during phage lytic growth. In Stx1 phages, the *stx1* genes have an additional promoter that is activated under iron-limiting conditions^4,5^, meaning Stx1 can be produced also in the absence of prophage induction. Nonetheless, high level production of both Stx1 and Stx2, and their subsequent release from bacteria relies on phage induction and phage-mediated bacterial lysis^6–8^.

As with other λ-like prophage, agents (e.g. quinolone antibiotics) that activate the host DNA damage response pathway (SOS response) induce the lytic growth of the Stx-encoding prophage^8^. Thus treatment of EHEC infections with quinolone antibiotics is contraindicated. While it is clear that transcriptional and translational inhibitors can be used to inhibit Stx production *in vitro*, several studies suggest that administration of any antibiotic increases the risk of severe EHEC-mediated disease^9,10^. It is also unknown whether Stx1/2 production can be blocked after the SOS response has already been induced (e. g. as a result of an accidental application of a quinolone antibiotic). Therefore we sought to determine if antibiotics that inhibit bacterial gene expression can be used to block Stx production either prior, or subsequent to activation of the host SOS system.

To answer these questions, we used a reporter gene-based test system that allows for time-resolved, simultaneous monitoring of the SOS response (*recA*P-*cfp*) and Stx1 production (*stx1::yfp*) in an EHEC O157:H7 EDL933 background. Our results show that only transcriptional and translational inhibitors, but not the cell wall synthesis inhibitor ampicillin, prevents Stx1 production *in vitro* following ciprofloxacin treatment, or prevents both, the ciprofloxacin induced SOS response and Stx1 production. Similar results were obtained when we analyzed the SOS response and *stx*1/2 transcription by RT-qPCR in a set of highly virulent clinical isolates. Thus, our results suggest that antimicrobials that inhibit the bacterial gene expression apparatus could be the basis for a causative therapy for treating EHEC infections.

## Results

### Construction of EHEC O157:H7 EDL933 Δstx2 stx1::yfp pMBM25

**Figure 1:**
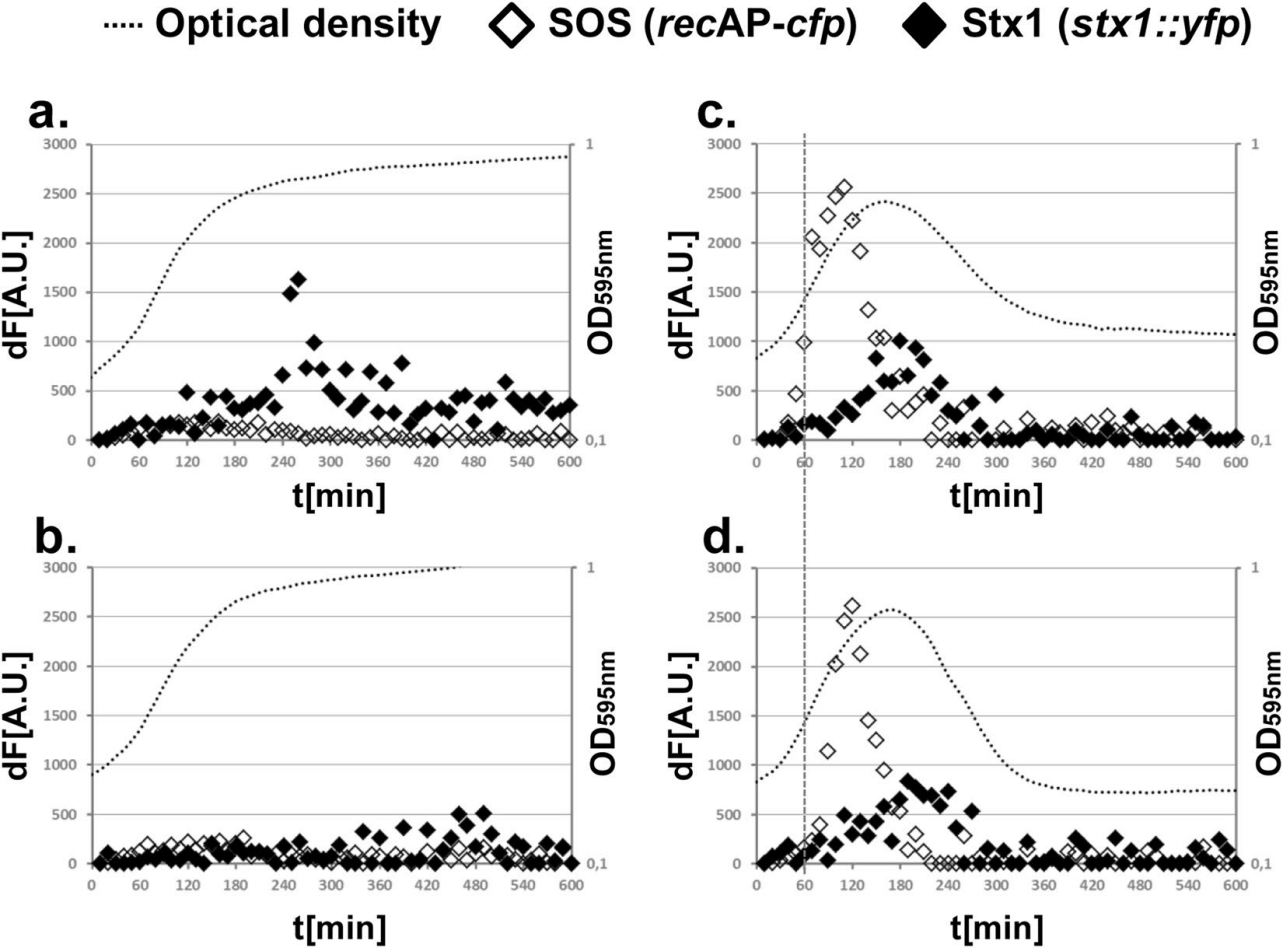
Kinetics of Stx1 expression and SOS response in EHEC O157:H7 EDL933. Shown are growth curves (black dots) and SOS response (*recA*P*-cfp*, open diamonds) and Stx1 expression (*stx1::yfp*, black diamonds) as increase of fluorescence over time (dF [A.U.]). (**a**) Cells were grown in M9 medium supplemented with casamino acids. (**b**) Growth in M9 medium and casamino acids, supplemented with 3 mM Fe_2_Cl_3_∙4H_2_O. (**c**) Cells were grown in M9 medium with casamino acids supplemented with 0.1 µg/ml of ciprofloxacin added at t = 0. (**d**) Growth in M9 medium and casamino acids supplemented with 0.1 µg/ml of ciprofloxacin added at t = 60 minutes.

In order to measure Stx1 production in the EHEC O157:H7 strain EDL933, we precisely replaced *stx1* by *yfp* (yellow fluorescent protein) in a Δ*stx2* derivative^11^. To simultaneously monitor SOS induction (see materials and methods for details)^12^, we also constructed a low copy plasmid containing a *recA* promoter (*recA*P) – *cfp* (cyan fluorescent protein) transcriptional fusion. During growth in minimal medium (Fig. 1a, black dots), the rate of increase in activity of the RecA CFP reporter fluorescence over time remained constant, indicating that the SOS response was not induced under these growth conditions (Fig. 1a, open diamonds). We found that the rate of Stx1 production, as monitored by YFP fluorescence (Fig. 1a, black diamonds), increased as the cells progressed through growth in log phase, showing a burst of expression around 260 min as the cells enter stationary phase. The increase of Stx1 levels was apparently due to the activation of the Fur dependent *E. coli* promoter of *stx1* and independent of the SOS response, as the increase in Stx1 levels was absent when the medium was supplemented with iron. Added iron did not alter the signal from the SOS reporter (Fig. 1b, black diamonds; Supplementary Fig. 1a)^13^.

In contrast, if the cells were grown in medium containing 0.1 µg/ml ciprofloxacin, the SOS response was induced immediately after dilution, with a peak in expression at around 100 min (Fig. 1c, open diamonds). Stx1 production was also activated by growth in ciprofloxacin (compare Fig. 1c and 1a), however the peak in Stx1 expression occurred approximately 80 minutes after the peak of SOS activity. As judged by the simultaneous drop in OD_595_, this peak in Stx1 expression occurred concomitantly with cell lysis. A similar pattern of SOS response and Stx1 production was observed when 0.1 µg/ml ciprofloxacin was added 60 min after the start of the experiment, but as expected the timing of the responses was delayed (compare Fig. 1c and 1d). The addition of a 10-fold lower concentration of ciprofloxacin (0.01 µg/ml) weakly induced the SOS reporter system (Supplementary Fig. 1b), but there was no apparent cell lysis. Nonetheless, it is possible that a small sub-population was still undergoing prophage induction (compare Supplementary Fig. 1b to Fig. 1a). Adding 0.001 µg/ml ciprofloxacin did not induce the SOS reporter system (compare Supplementary Fig. 1c,d to Fig. 1a).

### Kinetics of Stx1 accumulation and SOS response induction after antibiotic treatment

Activation of the SOS response induces the Stx1-encoding prophage and the overexpression of Stx1 during the late stages of phage lytic growth. Several classes of antibiotics exert their anti-bacterial effects by blocking gene expression. Consequently, we hypothesized that adding these types of antibiotics could block the production of Stx1 in cells which already have a fully induced SOS response. These antibiotics could also act to reduce overall basal amounts of Stx1 produced from the iron regulated *stx1* promoter of the prophage. In order to test these hypotheses, we compared the effect of adding antibiotics to cells grown in the presence or absence of SOS-inducing ciprofloxacin on the activity of our Stx1 and SOS reporters to the effect of adding ciprofloxacin alone. Based on the results shown in Fig. 1, we chose to investigate the effect of adding secondary antibiotics on Stx1 production at a fixed ciprofloxacin concentration of 0.1 µg/ml. We began by examining the effect of rifaximine, a transcription inhibitor that blocks the translocation of RNA polymerase^14^.

**Figure 2:**
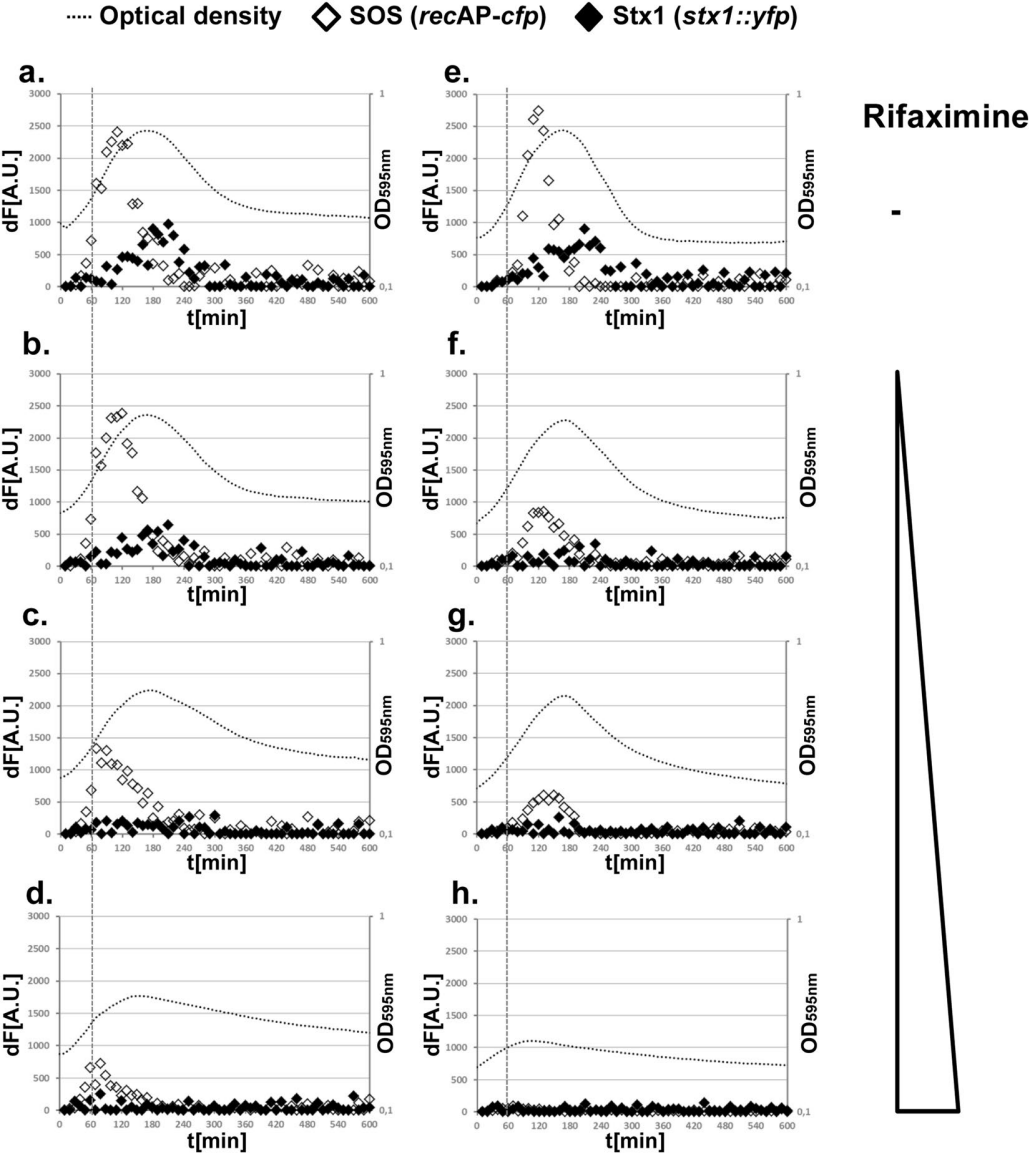
Rifaximine blocks Stx1 expression and SOS response in EHEC O157:H7 EDL933 in a concentration dependent manner. Shown are growth curves (black dots) and SOS response (*recA*P*-cfp*, open diamonds) and Stx1 production (*stx1::yfp*, black diamonds) as increase in fluorescence signal over time (dF [A.U.]). Cells were grown in M9 medium with casamino acids supplemented with 0.1 µg/ml of ciprofloxacin and at t = 60 min (**a**) H_2_O, (**b**) 1 µg/ml rifaximine, (**c**) 10 µg/ml rifaximine, (**d**) 100 µg/ml rifaximine was added. (**e**) Cells were grown in M9 medium with casamino acids alone, or supplemented with 1 µg/ml of rifaximine (**f**), 10 µg/ml of rifaximine (**g**) and with 100 µg/ml of rifaximine (**h**) followed by addition of 0.1 µg/ml ciprofloxacin at t = 60 min.

Whereas cells that were grown with 0.1 μg/mL ciprofloxacin alone displayed the induced SOS response and Stx1 reporters (Fig. 2a), adding 10 µg/ml rifaximine reduced the SOS reporter activity (compare open diamonds in Fig. 2c with Fig. 2a) and the production of Stx1 (compare black diamonds in Fig. 2c and Fig. 2a, ~200 min). Adding 100 µg/ml rifaximine to ciprofloxacin-treated cells immediately and almost completely blocked the expression of both reporter genes (Fig. 2d). We next examined whether the presence of rifaximine can prevent the ciprofloxacin-mediated induction of the SOS response. As described above, the SOS response was immediately induced when the cells were challenged with 0.1 µg/ml ciprofloxacin 60 min initiating growth (Fig. 2e).Importantly and in contrast to ciprofloxacin, rifaximine did *not* induce the SOS response over the entire range of rifaximine concentrations tested (compare open diamonds in Fig. 2e–h to Fig. 2a–d, before 60 min; Supplementary Fig. 2). Prior addition of 1 µg/ml rifaximine reduced the SOS-dependent increase in Stx1 production (compare Fig. 2f and Fig. 2e). Increasing the concentration of rifaximine further reduced the cells capacity to respond to ciprofloxacin (compare Fig. 2g to Fig. 2f) and at 100 µg/ml rifaximine no protein synthesis occurred, as judged by the lack in increase of fluorescence signals for both fluorophores (Fig. 2h).

### Antibiotics that inhibit transcription or translation stop and/or prevent SOS response and stx1 expression

**Figure 3:**
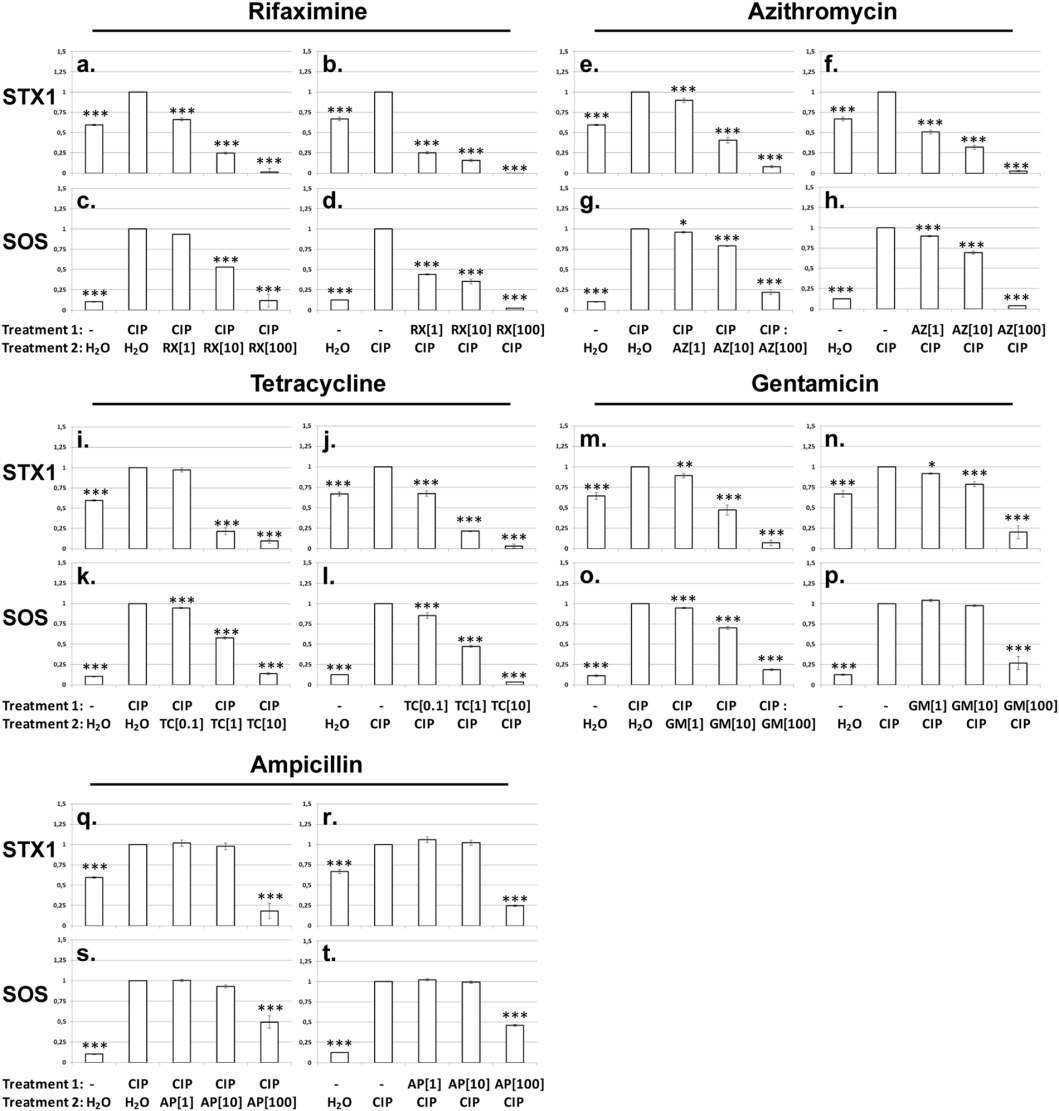
Stx1 expression and SOS response in EHEC O157:H7 EDL933 in response to different combinations of antibiotics normalized to the fully induced control (ciprofloxacin treatment). Shown is the ratio of Stx1 expression (total YFP) and SOS response (total CFP) of the indicated treatment in between 200 min and 60 min and Stx1 expression and SOS response of the fully induced control in the same time frame (ciprofloxacin treatment = 1).Treatment 1 indicates the composition of the medium at t = 0 min, treatment 2 indicates the addition of the second component 60 min after the beginning of the experiment. The ciprofloxacin concentration was kept constant at an f. c. of 0.1 µg/ml, the concentration of the second antibiotic is indicated in µg/ml in brackets. Shown are average ratios and standard deviations of three biological replicates. Ratios significantly different from the fully induced control are marked: *p < 0.05, **p < 0.01, ***p < 0.001. For details see text.

We next examined the ability of other antibiotics to affect SOS response and Stx1 production when added prior or subsequent to ciprofloxacin. As with rifaximine, we varied the order of antibiotic addition and the concentration of the “secondary” antibiotic. The translational inhibitors azithromycin, gentamicin and tetracycline all exhibited a concentration-dependent ability to block the SOS response and *stx1* expression, whether added after or before treating the cells with ciprofloxacin (Supplementary Figs. 3–6). We found that antibiotic addition altered only the amount of fluorescence produced by the SOS and Stx1 reporters; it did not affect the timing of these responses. To facilitate the comparisons between the effect of individual antibiotic treatments on SOS induction and Stx1 production, we normalized the total amount of fluorescence signal emitted by cells treated with ciprofloxacin and another antibiotic to the signal emitted by cells treated with ciprofloxacin alone (Fig. 3). In order to do so we calculated the total increase of fluorescence signal between 60 and 200 minutes after initiation of the experiment, as this timeframe corresponded to the time between addition of antibiotics and the peak of SOS induced Stx1 production and cell lysis (Fig. 2, Supplementary Figs. 3–6; for details see Materials and Methods). Rifaximine added either prior, or subsequent to the addition of 0.1 μg/mL ciprofloxacin, reproducibly reduced the SOS response signals to levels that were either similar, or lower than in the water control (Fig. 3c,d). Increasing concentrations of rifaximine also resulted in a progressive decrease in Stx1 reporter fluorescence. Adding 100 µg/ml of rifaximine reduced Stx1 reporter fluorescence to levels that are much lower than that seen in the water control (Fig. 3a,b). Very similar to adding rifaximine, we found that adding the translational inhibitors azithromycin (Fig. 3e–h), tetracycline (Fig. 3i–l) and gentamicin (Fig. 3m–p) each caused a concentration dependent reduction and/or a prevention of SOS induction. Similarly, a progressive decrease in Stx1 reporter fluorescence was observed, regardless of whether the antibiotic was added before, or after ciprofloxacin. We note that under our experimental conditions all the transcriptional and translational inhibitors were able to suppress Stx1 expression to a level that is at, or even below, the water control levels. In contrast, adding increasing concentrations of the cell wall synthesis inhibitor, ampicillin, did not result in a stepwise reduction in SOS response and/or Stx1 production, as expected (Fig. 3q–t). At 100 µg/ml ampicillin also this cell wall synthesis inhibitor caused a reduction of Stx1 production (Fig. 3q,r). However, as judged by reporter gene expression, the levels of SOS reporter produced in the time frame of the experiment were still higher than in the no treatment control (Fig. 3s,t). Notably we have also directly tested the effect of rifaximine and azithromycin on Stx1/2 production by EHEC O157:H7 EDL933 before and after ciprofloxacin treatment, with very similar results (Supplementary Fig. 7).

### Effect of antibiotic addition on SOS and stx gene transcription in other clinically important EHEC isolates

**Figure 4:**
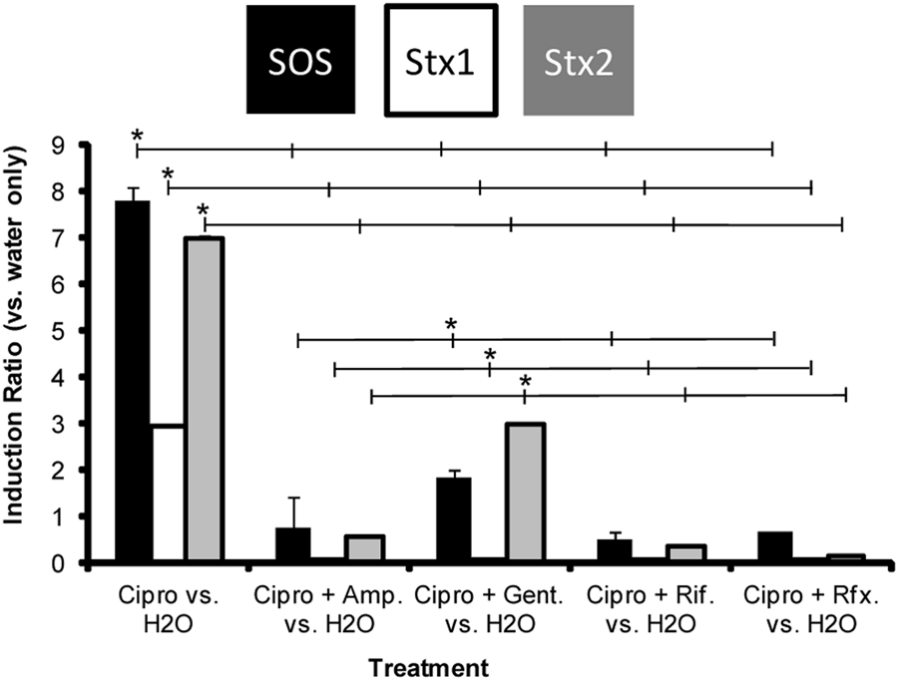
Effect of antibiotic addition on transcript levels of *umuD* (SOS response), *stx1* and *stx2* in EHEC O157:H7 EDL933. Shown is the relative effect of antibiotic treatment on *umuD* (black bars), *stx1* (white bars) and *stx2* (gray bars) transcript levels. These values were calculated by dividing the ΔΔC_T_ value for each treatment by the ΔΔC_T_ value found for each gene in the no treatment (H_2_O) control. ΔΔC_T_ value for each gene was determined by measuring the transcript level for *umuD*, *stx*1 and *stx*2 relative to the housekeeping gene *uidA*. For this measurement, RNA was isolated from cells grown in M9 medium supplemented with casamino acids without ciprofloxacin (Cipro) for 2 hr, with ciprofloxacin alone for 2 hr or with 0.1 μg/mL ciprofloxacin for 1 hr followed by growth in the presence of ampicillin (100 *μ*g/ml), gentamicin (Gent.)(50 *μ*g/ml), rifampicin (Rif.)(100 *μ*g/ml), or rifaximine (Rfx.) (100 *μ*g/ml) for an additional 1 hour, as indicated. Asterisks indicate statistically significant differences (p < 0.01) between ciprofloxacin-treated cells and all other treatments, as well as between ciprofloxacin + gentamicin treated cells and other cells treated with two antibiotics.

Having found that adding certain antibiotics can block ciprofloxacin-dependent induction of the synthesis of fluorescent reporter proteins that monitor SOS (RecA) and toxin production (Stx1), we wanted to verify these results in other clinically important strains. For these experiments, we used RT-qPCR to compare the effect of ciprofloxacin alone, or ciprofloxacin followed by the addition of ampicillin, gentamicin, rifampicin or rifaximine on the transcript levels of *umuD*, a gene in the SOS regulon, *stx*2 and/or *stx1*, relative to the housekeeping gene *uidA* in EHEC O157:H7 EDL933, the *stx*^+^ parent of our reporter strain. We found that two hours after adding 0.1 μg/mL ciprofloxacin, transcription of *umuD* in EHEC O157:H7 EDL933 increased by ~8-fold, as compared to water controls (Fig. 4, Supplementary Fig. 8). This finding is consistent with our reporter gene measurements showing that ciprofloxacin addition induces expression of genes in the SOS pathway (Fig. 1). We also found that ciprofloxacin addition to EHEC O157:H7 EDL933 increased the transcription of *stx1* by ~3-fold and *stx*2 by ~7-fold (Fig. 4, Supplementary Fig. 8). The greater relative effect of ciprofloxacin on *stx*2 transcription, as opposed to *stx1*, is consistent with the observation that expression of *stx2* in EHEC O157:H7 EDL933 is strictly dependent on the activation of the SOS regulon^4,5^. Subsequent addition of any type of secondary antibiotic reduced the level of ciprofloxacin-dependent stimulation of *umuD*, *stx1* and *stx*2 transcription (Fig. 4, Supplementary Fig. 8). As compared to the translation inhibitor gentamicin, the transcriptional inhibitors had the greater effect on the *umuD*, *stx1* and *stx*2 transcript levels, reducing them to levels that are not significantly different (*p* ≤ 0.05) from those observed in water only controls. However regardless of mechanism of action, all secondary antibiotics tested, substantially reduced (≥3-fold) the effect of adding ciprofloxacin on *umuD*, *stx1* and *stx*2 transcript levels.

**Figure 5:**
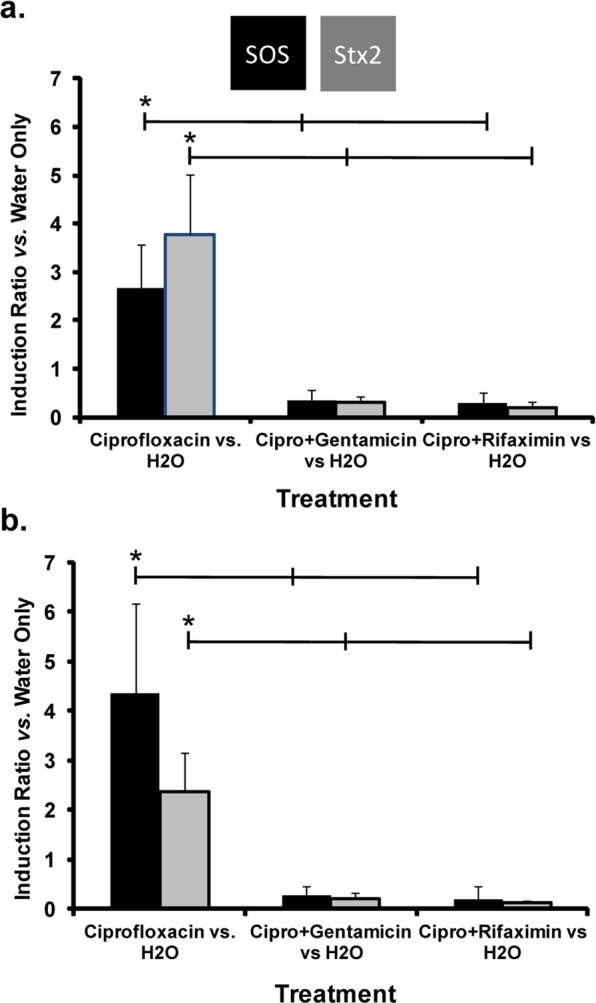
Effect of antibiotic addition on transcript levels of *umuD* (SOS response), and *stx2* in EHEC HUSEC-1 and HUSEC-41. Shown is the relative effect of antibiotic treatment on *umuD* (black bars) and *stx2* (gray bars) transcript levels. These values were calculated by dividing the ΔΔC_T_ value for each treatment by the ΔΔC_T_ value found for each gene in the no treatment (H_2_O) control. ΔΔC_T_ value for each gene was determined by measuring the transcript level for *umuD* and *stx*2 relative to the housekeeping gene *uidA*. For this measurement, RNA was isolated from cells grown in M9 medium supplemented with casamino acids without ciprofloxacin for 2 hr, with ciprofloxacin (Cipro) alone for 2 hr or with 0.1 μg/mL ciprofloxacin for 1 hr followed by growth in the presence of gentamicin (Gent.) (50 *μ*g/ml), or rifaximine (Rfx.) 100 *μ*g/ml for an additional 1 hour, as indicated. Asterisks indicate statistically significant differences (p < 0.01) between ciprofloxacin-treated cells and all other treatments.

We also examined the effect of antibiotic treatment on SOS and Stx2 production in two HUS-associated non-O157 EHEC strains, HUSEC-1, an O111:H10 strain and HUSEC-41, an O104:H4 strain that is very closely related to the strain that was responsible for a massive EHEC outbreak in Germany in 2011^15,16^. We chose to examine these two HUSEC strains because the immunity regions and Stx2-synthesis control regions of these phage are 99% identical at the nucleotide level, but are different from those of EHEC O157:H7 EDL933 and are integrated in different strain backgrounds. Nonetheless, when the two non-O157:H7 HUSEC EHEC strains were grown in the absence of antibiotics for 2 h, the amounts of *umuD* and *stx2* transcripts increased, very similar to what we have observed in EHEC O157:H7 EDL933 (Fig. 5a,b, Supplementary Fig. 9a,b). Adding ciprofloxacin to the HUSEC-1 and HUSEC-41 strains increased the transcription of *umuD* by ~2.3- and ~4.3-fold, respectively, as compared to water controls (Fig. 5a,b, Supplementary Fig. 9a,b). Ciprofloxacin addition also increased transcription of the *stx2* gene in both these strains by similar amounts (~2.5- to 3.5-fold). The amounts of *umuD* and *stx2* transcripts that accumulated in response to ciprofloxacin treatment were higher in HUSEC-41 than in HUSEC-1. Similar to our findings with EHEC O157:H7 EDL933, adding either a transcription inhibitor (rifaximine), or a translational inhibitor (gentamicin), to HUSEC-1 and HUSEC-41 strains that were first grown for 60 minutes with ciprofloxacin, blocked the effect of ciprofloxacin on *umuD* and *stx*2 transcript amounts in both these strains (Fig. 5a,b, Supplementary Fig. 9a,b). HUSEC-1 and HUSEC-41 are resistant to ampicillin so we did not perform experiments with that antibiotic. Again mirroring the results with EHEC O157:H7 EDL933, the addition of rifaximine or gentamicin subsequent to the addition of ciprofloxacin resulted in *umuD* and *stx*2 transcript levels that were not statistically different from those seen with the water only control (Fig. 5a,b, Supplementary Fig. 9a,b).

## Discussion

Others have already shown that antibiotics that inhibit transcription or translation also inhibit Stx1/2 production in various genetic backgrounds and experimental conditions^6,17–20^. However, we show here for the first time that such antibiotics can be used to block *stx1/2* expression after the SOS response was fully induced and if administered before a DNA damaging agent they can block, *in vitro*, both the SOS response and Stx1/2 production. In addition to facilitating studies of the effects of antibiotics on SOS induction and Stx production, our fluorescent reporter gene based assay allowed us to examine the kinetics of these processes at high temporal resolution. For example, our results show that the timing of cell lysis and Stx1 production upon ciprofloxacin addition are very similar to those described by others for mitomycin C. That is, both cell lysis (starting around 180 min) and the peak in Stx1 production (around 200 min) were substantially delayed with respect to the peak of the SOS response, which occurred around 120 min after the beginning of the experiment (Fig. 1c). This observation suggested that Stx1 production can be blocked even after the addition of an SOS inducer. Consistent with this suggestion, we find that adding the transcription inhibitor rifaximine, or the translation inhibitors azithromycin, tetracycline or gentamicin, blocked Stx1 production in a concentration dependent manner, but adding the cell wall synthesis inhibitor ampicillin did not (Fig. 3). Notably in our experimental setup, the amounts of rifaximine, azithromycin, tetracycline and gentamicin were substantially lower, relative to their minimal inhibitory concentration than the amounts of ampicillin (Supplementary Table 1). Thus, the inhibition of Stx1 production is indeed a direct result of their mechanism of action. Notably, this is not only true for the YFP reporter, but also for the wild type Shiga toxins of EHEC O157:H7 EDL933 (Supplementary Fig. 7). These results may therefore inform the development of potential therapeutic regimes.

Our results suggest that bacterial gene expression inhibitors, if applied after an accidental uptake - e.g. in the case that the prescription preceded the diagnosis of the disease - of an SOS response inducing antibiotic could be used to keep Stx1 (Fig. 3) and/or Stx2 (Fig. 5) at or even below no treatment levels. Moreover, as judged by the sudden drop in the SOS response reporter expression, the highest concentrations of the transcriptional and translational inhibitors are able to rapidly stop the production of functional protein (Fig. 2; Supplementary Figs. 3–6). Thus our data shows that antibiotics that inhibit bacterial gene expression can block Stx1/2 production after the bacterial SOS response is induced. Moreover, this finding also shows that with respect to Stx production, administration of these antibiotics can completely prevent SOS response induction and Stx1 production when applied as first antibiotic (Fig. 3; compare Stx1 and SOS response at the highest antibiotic concentrations to the water control).

Intoxication with Stx can result in the life-threatening HUS. High level production and release of Stx by EHEC strains requires induction of the Stx-encoding prophage resident in all EHEC strains. The current guideline that all antibiotics should be avoided in life-threatening EHEC infections^10^ apparently derives from the suggestion that such treatments may increase the risk of HUS development by activating the prophage via the bacterial SOS response. However many classes of antibiotics; e.g. macrolides^6,21–23^, carbapenems^6,17^, aminoglycosides^6,24^, rifampin^6^, rifaximine^17^, and fosfomycin^6^, have either no, or a suppressive effect on Stx production. Consistent with the mechanism of action of these antibiotics, we find that addition of a secondary, suppressive antibiotic either prior or subsequent to addition of the SOS-inducing antibiotic ciprofloxacin blocks induction of the SOS response in EHEC. Consequently, these treatment regimens also block production of Stx1 and/or Stx2. These observations along with clinical data showing beneficial effects of antibiotics in the course of EHEC O104:H4 infections^25^ lead to the suggestion that antibiotic therapy or particular combinations of antimicrobials may be of value in treating EHEC infection in humans^26^.

Our results suggest that in particular the use of antibiotics that inhibit the bacterial transcription and/or translation may be useful as a causative therapy for EHEC infections. For example, rifaximine was shown to reach up to 80 fold higher concentrations in feces during standard oral therapy than the highest concentration that was used in this study^27^. In addition, rifaximine was shown to be very effective in the treatment of infections with enteroaggregative *E. coli*^28^. Therefore rifaximine appears to be a very promising candidate for the treatment of EHEC infections as well and we believe that this should be evaluated in clinical trials in the future.

## Materials and Methods

### Bacterial strains and plasmids used in this study

All bacterial strains used in this study are listed in the Supplementary Table 2. The details of the construction of the reporter strain and plasmid are described in supplementary materials and methods.

### Growth conditions and reporter gene expression measurements

All reporter gene based, time resolved measurements were done M9 supplemented with 0.4% glucose, 0.4% casamino acids and 12.5 µg/ml kanamycin (standard medium), which was the basic medium composition for all experiments. The time-resolved measurements were performed in a Tecan infinite 200pro instrument at 37 °C. Further details are described in supplementary materials and methods.

### RT-qPCR analysis of Stx1, Stx2 and SOS gene expression

RT-qPCR following RNA extraction was done using a reaction mixture using the Fast SYBR^TM^ Green Master Mix (Applied Biosystems) in Bio-Rad iQ5 real-time PCR detection system. The details are described in supplementary materials and methods.

### Statistical methods

The data presented are derived from multiple experimental replicates (≥3). For the RT-qPCR analysis, each replicate comprised ≥3 technical replicates. Analysis of variance (ANOVA) of the resulting data was used to assess the significance of the differences between sample groups. Pairwise comparisons between groups were performed using t-tests with corrections for multiple testing using the Holm method.

## Supplementary information

Supplementary Info
