# Supplementary material for: Transcriptional and Translational Inhibitors Block SOS Response and Shiga Toxin Expression in Enterohemorrhagic Escherichia coli
Source: Sci Rep. 2019 Dec 11;9:18777. doi: 10.1038/s41598-019-55332-2 (PMC6906329; doi:10.1038/s41598-019-55332-2)
Supplement: Supplementary file 1 — Supplementary Info [file 41598_2019_55332_MOESM1_ESM.pdf]

Transcriptional and Translational Inhibitors block SOS response and Shiga Toxin Expression in Enterohemorrhagic *Escherichia coli*

Michael Berger[1*], Iqbal Aijaz[2], Petya Berger[1], Ulrich Dobrindt[1], Gerald Koudelka[2]

[1]Institute of Hygiene, University of Münster, Münster, Germany

[2]Department of Biological Sciences, University at Buffalo, Buffalo, USA

*Correspondence: michael.berger@ukmuenster.de

Supplementary data

Supplementary Materials and Methods

Bacterial strains and plasmids used in this study

EHEC O157:H7 EDL933 *Δstx1/2 stx1::yfp* was constructed by allelic replacement of the kanamycin resistance cassette in EHEC EDL933 O157:H7 *Δstx1/2* by an *yfp-cat* cassette generated with the primers MBP 224/225 (Supplementary Table 3) and pMB54 as template[1-3]. The fusion of the 5´ UTR of *stx1* to *yfp*-cat was checked by PCR over the 5' and 3' junctions of the chromosome and the reporter module with the primers MBP 228/206 and MBP 254/5, respectively. The PCR product over the 5´ junction was additionally analyzed by Sanger sequencing in order to exclude any point mutations. The phage carrying the *stx1::yfp-cat* allele could lysogenize *E. coli* K-12 MG1655, indicating that it was fully functional. For the construction of pMBM25 we first created a chromosomal *recA* promoter (*recA*P)-*cfp* fusion using a PCR product generated with primers MBP 262/263 and pMB47 (*cfp-aph(3')-Ia*) as template for RedE/T recombineering[1]. Afterwards the *recA*P-*cfp-aph(3')-Ia* module was amplified from chromosomal DNA using the primers MBP 265/266, digested with *KpnI* and cloned in the *FspI* and *KpnI* digested low-copy plasmid pWKS30[4]. Plasmid pWKS30 was chosen as cloning vector because it contains a pSC101 origin of replication which does not change the copy number in response to UV irradiation and therefore does not interfere with the SOS system[5]. Afterwards the plasmid was isolated, the correctness of the *recA*P-*cfp* junction confirmed by Sanger sequencing with primer MBP 185 and afterwards named pMBM25. Transformation of pMBM25 into EHEC O157:H7 EDL933 *Δstx1/2 stx1::yfp* resulted in strain EHEC O157:H7 EDL933 *Δstx1/2 stx1::yfp pMBM25* that was used to indirectly monitor SOS-response and Stx1 production over time by reporter gene expression measurement.

Growth conditions and reporter gene expression measurements

For the reporter gene based indirect measurements of Stx1 (YFP) and SOS response (RecA, CFP), EHEC O157:H7 EDL933 *Δstx1/2 stx1::yfp pMBM25* was plated on LB agar plates containing 12.5 µg/ml

kanamycin for plasmid maintenance and incubated at 37 °C overnight. The next day single colonies were inoculated in 2 ml M9 supplemented with 0.4 % glucose, 0.4 % casamino acids and 12.5 µg/ml kanamycin (standard medium), which was the basic medium composition for all experiments, except if otherwise noted and incubated at 37°C and 180 rpm overnight in an Infors HT Multitron Standard incubator (Infors, Einsbach, Germany). The next day, the cells were diluted 1 to 10 in standard medium (150 µl f. V.) without the tested antibiotic in black µ-clear plates with transparent bottom and lid (Greiner Bio-One, Frickenhausen, Germany) and incubated in a Tecan infinite 200pro instrument at 37 °C. Each measurement cycle of the experiments started with linear shaking at an amplitude of 1 mm for 5 seconds after which optical density at 595 nm (OD_{595nm}), CFP signal (excitation at 442 nm /emission at 482 nm / gain 85) and YFP signal (excitation at 514 nm /emission at 550 nm / gain 100) were automatically recorded. Afterwards the cells were incubated with linear shaking at an amplitude of 2 mm for 450 seconds before the next measurement cycle started. Exactly 60 min after the start of the experiment, 1.5 µl of the second antibiotic was added to the indicated final concentration, 1.5 µl of water served as negative control and the measurement was continued with the settings described above. For the time resolved measurements, the production of the protein was calculated as increase of fluorescence signals (F [A.U.]) over time with dF_n [A.U.] = $F_n - F_{n-1}$. Negative values for dF_n [A.U.] were set to zero and the values for the weaker YFP signal were multiplied by a factor of 10 for a convenient representation in one graph with the CFP values. The normalized relative SOS response and Stx1 production was calculated by $F_{response}$= F $[A.U.]_{200min}$ – F $[A.U.]_{60min}$ for each treatment and then normalizing the response to the fully induced control ($F_{response}$ ciprofloxacin = 1) for each biological replicate. The graphs show average values and standard deviations for the normalized relative SOS response and Stx1 production for three biological replicates. The concentrations of both CFP and YFP under standard conditions w/o iron were calculated by normalizing background corrected CFP and YFP signals to blank corrected OD_{595nm}, as described previously[3].

RT-qPCR analysis of Stx1, Stx2 and SOS gene expression.

EHEC O157:H7 EDL933, EHEC O111:H10 HUSEC-1 and, an EHEC O104:H4 HUSEC-41 were maintained on LB agar plates. Singles colonies of each strain were inoculated in M9 supplemented with 0.4%glucose, 0.4% casamino acids and grown with shaking to saturation (~10^8/ml) at 37°C. The overnight cultures were diluted 50-fold into the same fresh medium with or without 0.1 μg/ml ciprofloxacin. The cultures were incubated at 37 °C while shaking for 60 min. At this stage, the second antibiotic(s) or water were added. When used, the second antibiotic was added at final concentrations of ampicillin 100 μg /ml, gentamicin 50 μg /ml, rifampicin 100 μg /ml, and rifaximin 100 μg /ml. At 120 min after start of the experiment, the bacterial cells were harvested by centrifugation at 14,000 g. The supernatant was discarded and the resulting pellet was frozen in dry ice and processed immediately to minimize RNA degradation.

Total RNA was extracted via hot-phenol method. Briefly, the pellet was resuspended in 500 μl TES (10 mM Tris pH 7.6, 1mM EDTA, 0.5% SDS) followed by the addition of equal volume of acid phenol:chloroform. The tubes were maintained at 65 °C for 10 minutes. The tubes were mixed by inverting 4-5 times each minute during incubation. Subsequently, the samples were centrifuged at 14,000 g at 4°C for 10 minutes, and the aqueous phase was removed to a clean tube. Any residual acid phenol:chloroform was removed via two subsequent chloroform extractions. The total RNA was precipitated via ethanol precipitations and its concentration was determined by measuring A_{260} using a NanoDrop 2000 UV spectrophotometer (Thermo Scientific).

For cDNA synthesis 1 μg of total RNA was digested using RQ1 RNase-Free DNase (Promega Corporation) following manufacturer’s protocol. RNA was reverse transcribed using RevertAid Reverse Transcriptase (Thermo Scientific) following manufacturer’s instructions. In brief, 0.5 μg of total RNA and 2 μl of random hexamer primer (0.2 μg/μl) were mixed together and incubated at 65° C for 5 min, chilled on ice, briefly centrifuged and placed back on ice. Subsequently, the following reagents were added in order: 1 μl RNase inhibitor, 4 μl 5× RT buffer, 2 μl dNTP, 2 μl random

hexamer, and 1 μl of RevertAid Reverse transcriptase. The reaction mixture was incubated at 42 °C for 1 hour followed by an incubation at70 °C for 10 min to terminate the reaction.

cDNA was amplified as a 20 µl reaction mixture using the Fast SYBR^{TM} Green Master Mix (Applied Biosystems) in Bio-Rad iQ5 real-time PCR detection system. We used the following two-step thermal profile: 5 min at 95°C, 45 repeats of 10 s at 95°C, 45 s at 60°C. The relative quantification method (*vs uidA*) was used to measure change in gene expression in response to treatment. The standard curves for qPCR were generated using genomic DNA and the genomic DNA was extracted using InstaGene matrix (Bio-Rad, Hercules, CA, USA) following the manufacturer's protocol. The primers and targets are listed in Supplementary Table 3.

Stx1 /Stx 2 detection in culture supernatants of EHEC O157:H7 EDL933

EHEC O157:H7 EDL933 was plated on LB agar plates and incubated overnight at 37°C. The next day, 5 individual colonies were inoculated in 2 ml M9 supplemented with 0.4 % glucose, 0.4 % casamino acids and incubated at 37°C and 180rpm overnight in an Infors HT Multitron Standard incubator (Infors, Einsbach, Germany). The next day, the overnight cultures were mixed in equal amounts. Afterwards the mixed overnight cultures were diluted 1:10 in glass tubes containing M9 supplemented with 0.4 % glucose, 0.4 % casamino acids, or the same medium containing ciprofloxacin, rifaximine or azithromycin at the indicated concentration (treatment 1, supplementary figure 7). After 1 h at 37°C and 180rpm, either none (H_2O), or the secondary antibiotic was added to the indicated concentration (treatment 2, supplementary figure 7) and the incubation was continued overnight. The next day, 1 ml of the culture was removed, centrifuged and the supernatant was filter sterilized. Afterwards we used the Ridascreen® Verotoxin Kit according to the manufacturer´s instructions for the immunological detection of Stx1 /2 in a dilution series of the culture supernatants (1:1; from row A to row H for each sample). The culture supernatant of *E. coli* K-12 MG1655 grown in the presence of 0.1 µg/ml ciprofloxacin served as negative control (MG1655). The positive control

(+) was provided together with the kit (supplementary figure 7 A). Absorbance at 450 nm was measured in a Tecan infinite 200pro instrument and the fold differences in the signals in comparison to the uninduced control were calculated. For the uninduced control, as well as for the combined treatments (CIP/RX, CIP/AZ, RX/CIP, AZ/CIP) the absorbance values from row B were used, for the CIP/ H_2O treatment the absorbance value of row F was used for the calculations (supplementary figure 7 B, C).

Determination of minimal inhibitory concentration (MIC)

In order to determine the MICs of the antibiotics used in our experimental setup, EHEC O157:H7 EDL933 *Δstx1/2 stx1::yfp pMBM25* was plated on LB agar plates containing 12.5 µg/ml kanamycin for plasmid maintenance and incubated at 37 °C overnight. The next day, 5 single colonies were inoculated in 2 ml M9 supplemented with 0.4 % glucose, 0.4 % casamino acids and 12.5 µg/ml kanamycin and incubated overnight at 37 °C and 180 rpm in an Infors HT Multitron Standard incubator (Infors, Einsbach, Germany). The next day, the overnight cultures were mixed in equal amounts. Afterwards the mixed overnight cultures were diluted 1:200 in microtiter plates containing M9 supplemented with 0.4 % glucose, 0.4 % casamino acids and a dilution series of the tested antibiotic in the same medium. Afterwards the plates were incubated overnight at 37°C and the next day growth was assessed by measuring the OD_{595nm} in a Tecan infinite 200pro instrument. The MIC was defined as the lowest concentration of the antibiotic at which no growth was observed (Supplementary Table 1).

Supplementary Figures

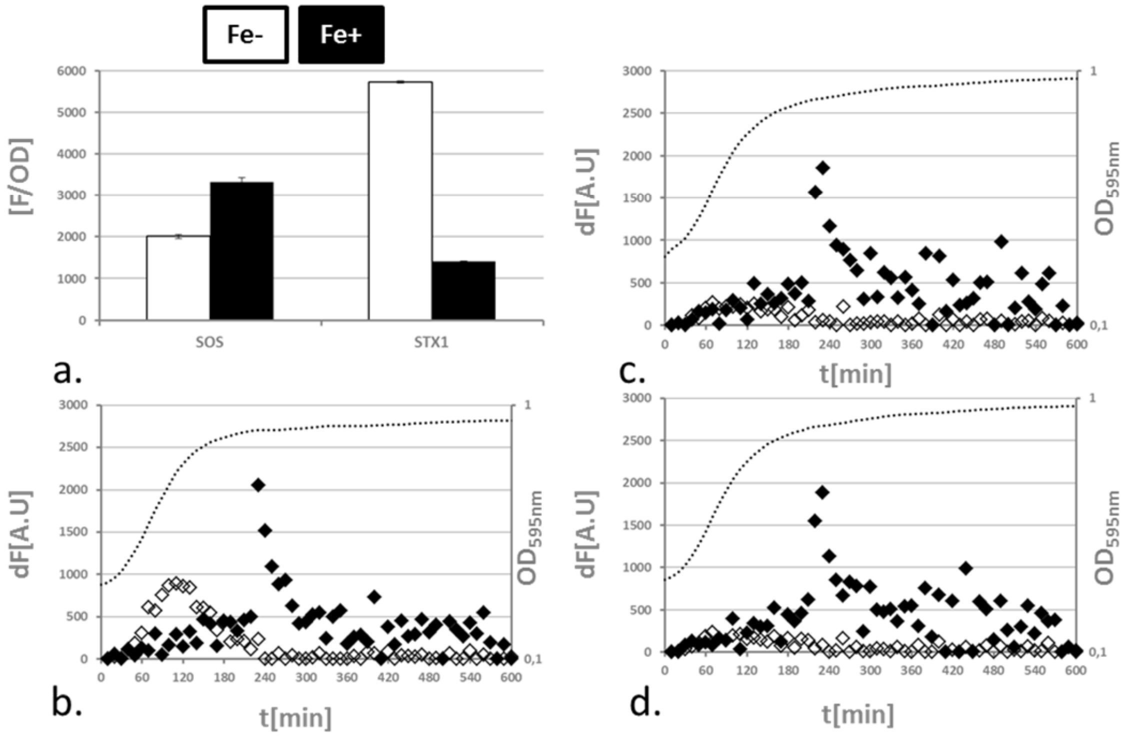

Supplementary Figure 1. SOS response and Stx1 expression in EHEC O105:H7 EDL933: Iron and ciprofloxacin concentration. (a) Whereas the SOS response is slightly elevated in the presence of iron in the medium, the overall amount of Stx1 is approximately 5 fold reduced in overnight cultures. Shown are average values and standard deviations in SOS response and Stx1 signals normalized to optical density in overnight cultures of three biological replicates. **(b-d)** Shown are growth curves (black dots) and SOS response (*recA*P-*cfp*, open diamonds) and Stx1 expression (*stx1::yfp*, black diamonds) as increase in fluorescence signal over time (dF [A. U.]). **(b)** Whereas the cells still show a weaker, but detectable SOS response to 0.01 µg/ml ciprofloxacin, but do not lyse anymore (compare to growth curve in figure 1 c), there was no detectable SOS response at 0.001 µg/ml ciprofloxacin **(c)** or at 0.0001 µg/ml ciprofloxacin **(d)** and the SOS response and Stx1 expression pattern resembled the growth phase dependent expression patterns (compare b and c to figure 1 a).

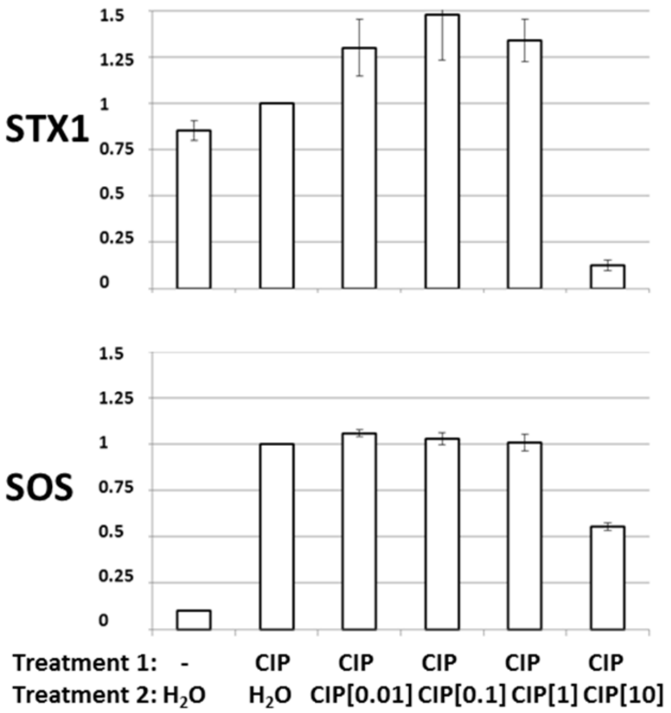

Supplementary Figure 2. Ciprofloxacin as first and secondary antibiotic. Note the further increase of Stx1 (from 0.01 to 1 µg/ml additional ciprofloxacin). Only at 10 µg/ml ciprofloxacin a reduction of Stx1 below the no treatment control can be observed. In contrast to rifaximine, azithromycin and azithromycin and tetracycline, but similar to ampicillin, there is no step-wise reduction Stx1. Similarly, the SOS response does not reach levels at, or below the no treatment control.

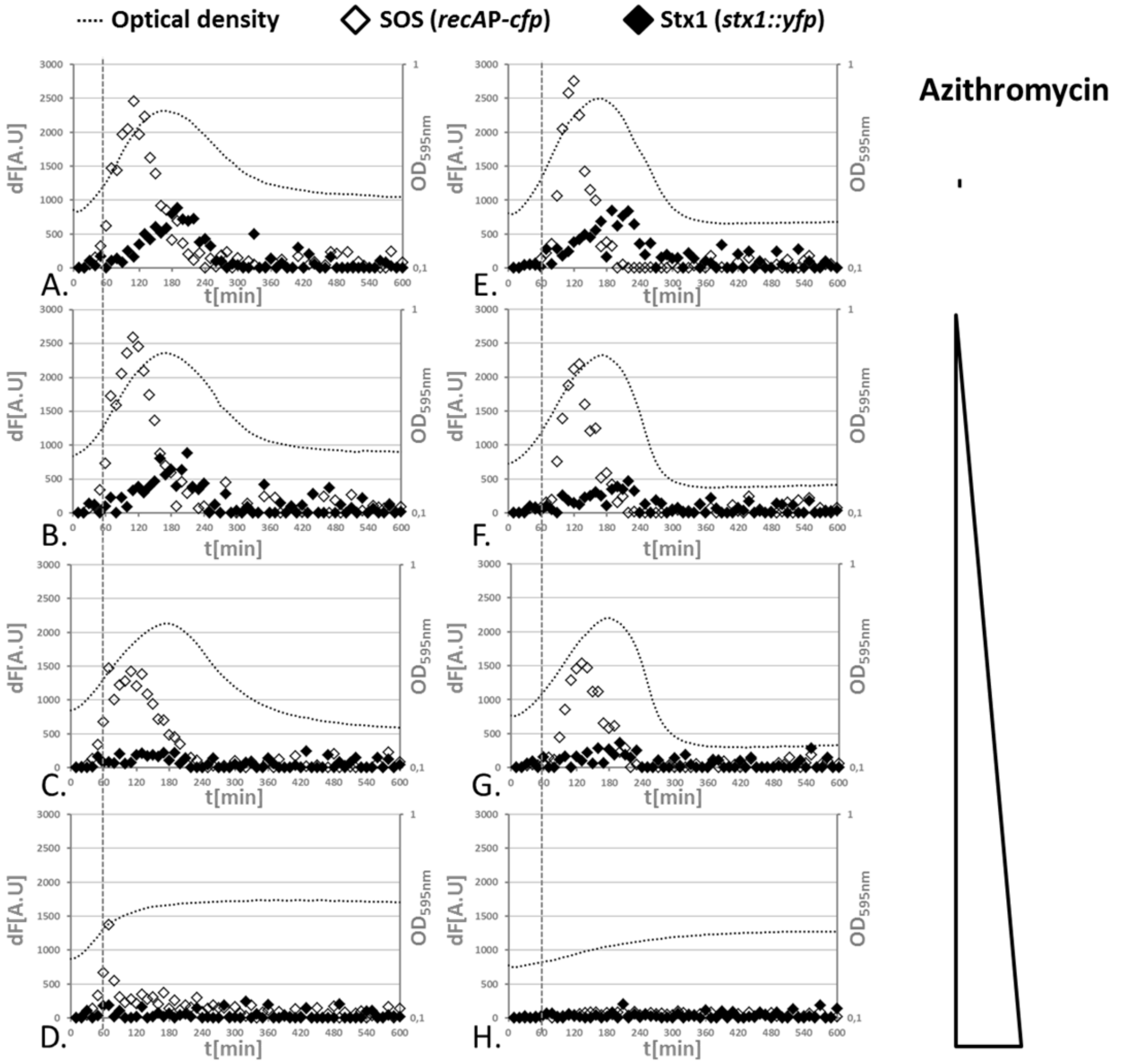

Supplementary Figure 3. Azithromycin blocks SOS response and Stx1 expression in EHEC O105:H7 EDL933 in a concentration dependent manner. Shown are growth curves (black dots) and SOS response (*rec*AP-*cfp*, open diamonds) and Stx1 expression (*stx1::yfp*, black diamonds) as increase of fluorescence over time. **(A-D)** Azithromycin (grey dashed line) can block Stx1 expression after the SOS response was induced by diluting the cells in medium containing 0.1 µg/ml ciprofloxacin **(A).** Whereas adding azithromycin to an f. c. of 1 µg/ml has little effect on SOS response and Stx1 expression **(B)**, 10 µg/ml azithromycin reduces both SOS response and Stx1 expression **(C)** and an f. c. of 100 µg/ml azithromycin stops SOS response and consequently Stx1 expression immediately **(D)**. **(E-D)** Azithromycin can prevent SOS response and Stx1 expression induction by 0.1 µg/ml ciprofloxacin (grey dashed line). If the medium contains azithromycin from the beginning of the experiment, the SOS response is not induced (compare to A-D). Whereas a slight induction of the SOS response can still be detected if the medium contained 1 µg/ml azithromycin **(F)** and further reduced at 10 µg/ml **(G)** and a complete block of the capacity of the cells to induce the SOS response is observed at 100 µg/ml azithromycin **(H)**. Consequently, Stx1 expression is completely abolished at 100 µg/ml azithromycin.

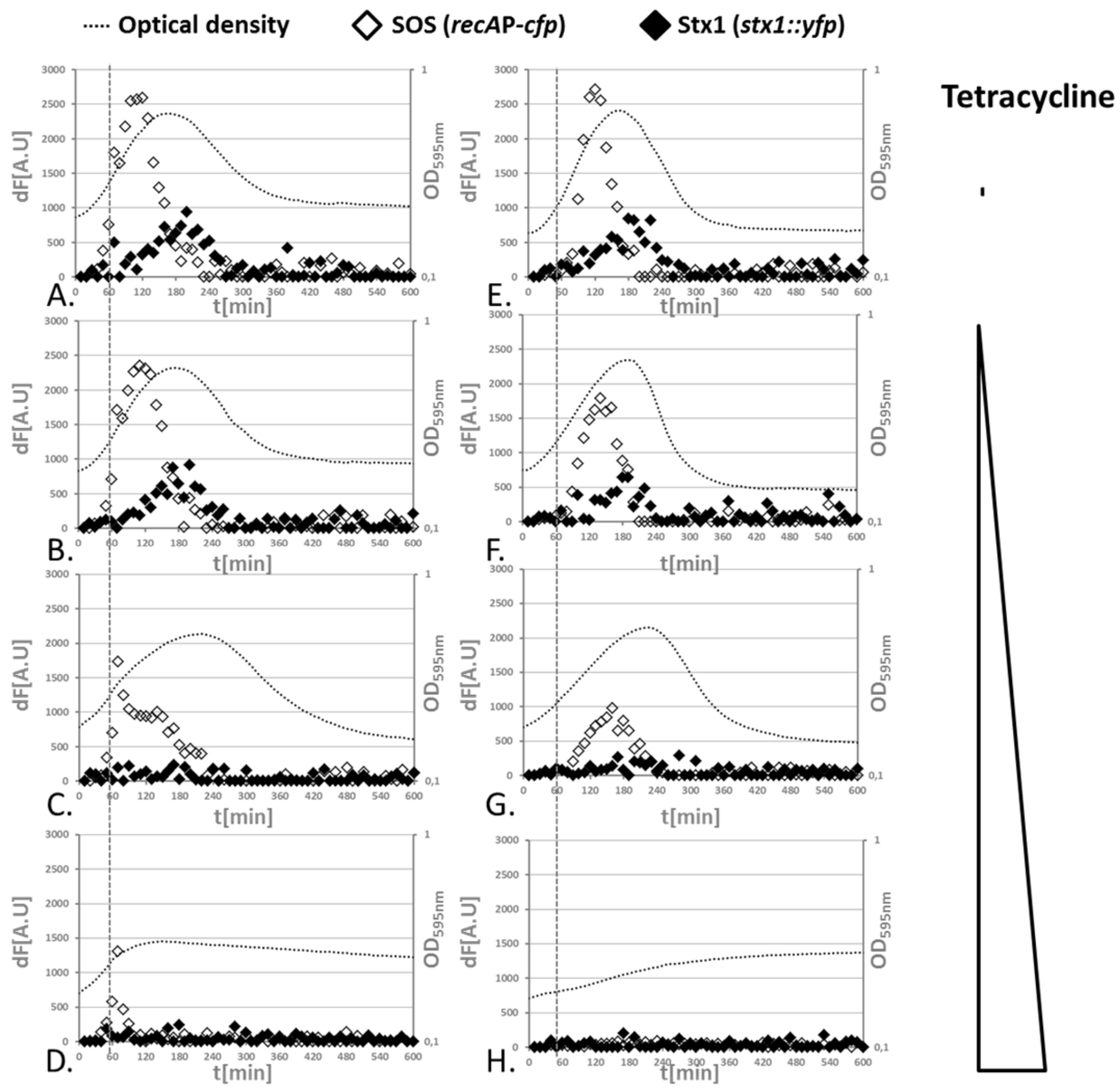

Supplementary Figure 4. Tetracycline blocks SOS response and Stx1 expression in EHEC O105:H7 EDL933 in a concentration dependent manner. Shown are growth curves (black dots) and SOS response (*recA*P-*cfp*, open diamonds) and Stx1 expression (*stx1::yfp*, black diamonds) as increase of fluorescence over time. **(A-D)** tetracycline (grey dashed line) can block Stx1 expression after the SOS response was induced by diluting the cells in medium containing 0.1 µg/ml ciprofloxacin **(A).** Whereas adding tetracycline to an f. c. of 0.1 µg/ml has little effect on SOS response and Stx1 expression **(B)**, 1 µg/ml tetracycline reduces both SOS response and Stx1 expression **(C)** and an f. c. of 10 µg/ml tetracycline stops SOS response and consequently Stx1 expression immediately **(D)**. **(E-D)** tetracycline can prevent SOS response and Stx1 expression induction by 0.1 µg/ml ciprofloxacin (grey dashed line). If the medium contains tetracycline from the beginning of the experiment, the SOS response is not induced (compare to A-D before 60 min). Whereas an induction of the SOS response can still be detected if the medium contained 0.1 µg/ml tetracycline **(F),** it is reduced at 1 µg/ml **(G)** and a complete block of the capacity of the cells to induce the SOS response is observed at 10 µg/ml tetracycline **(H)**. Consequently, Stx1 expression is completely abolished at 10 µg/ml tetracycline.

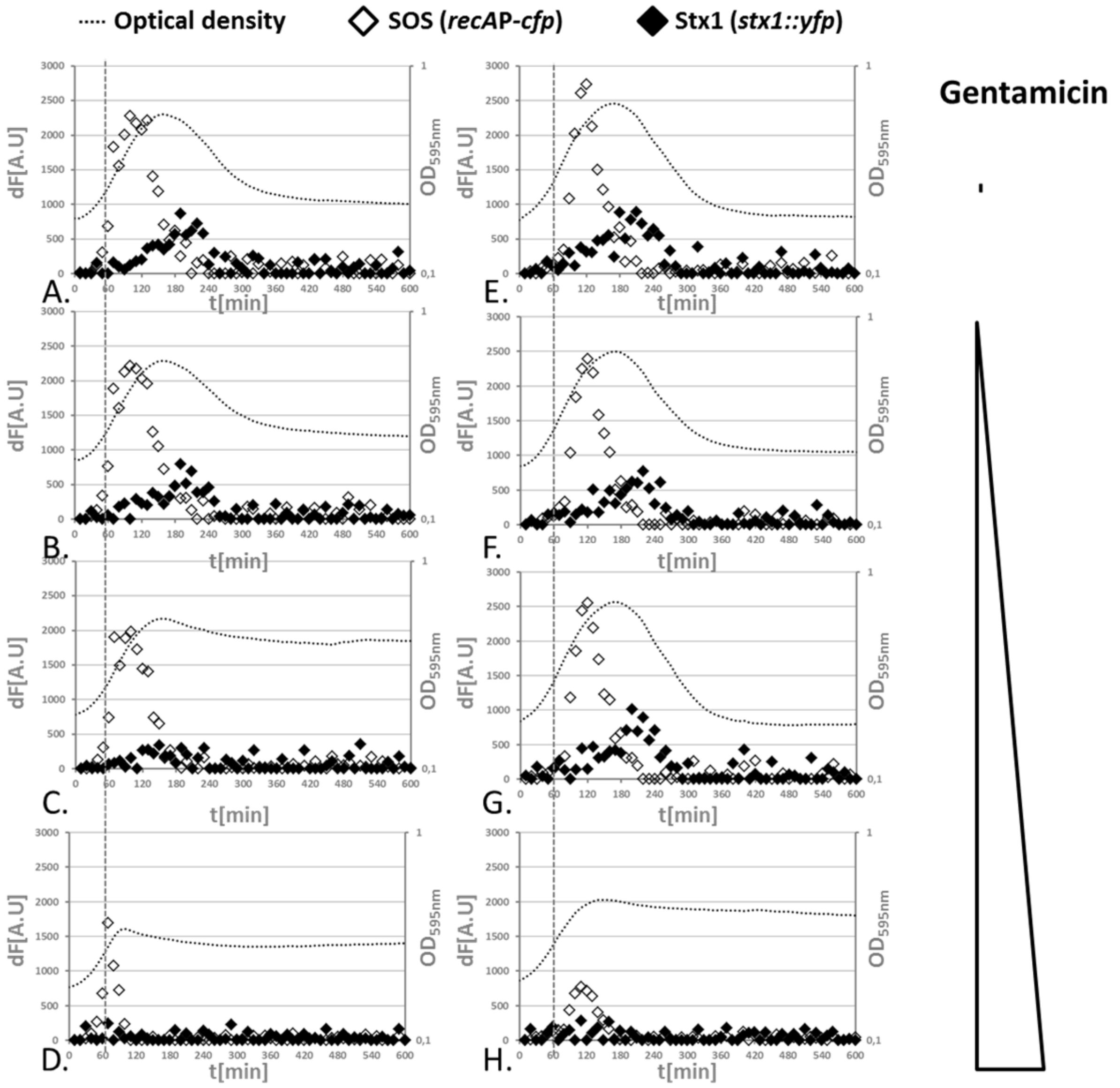

Supplementary Figure 5. Gentamicin blocks SOS response and Stx1 expression in EHEC O105:H7 EDL933 in a concentration dependent manner. Shown are growth curves (black dots) and SOS response (*recA*P-*cfp*, open diamonds) and Stx1 expression (*stx1::yfp*, black diamonds) as increase of fluorescence over time. **(A-D)** gentamicin (grey dashed line) can block Stx1 expression after the SOS response was induced by diluting the cells in medium containing 0.1 µg/ml ciprofloxacin **(A).** Whereas adding gentamicin to an f. c. of 1 µg/ml has little effect on SOS response and Stx1 expression **(B)**, 10 µg/ml gentamicin reduces both SOS response and Stx1 expression **(C)** and an f. c. 100 µg/ml gentamicin stops SOS response and consequently Stx1 expression immediately **(D)**. **(E-D)** gentamicin can prevent SOS response and Stx1 expression induced by 0.1 µg/ml ciprofloxacin (grey dashed line). If the medium contains gentamicin from the beginning of the experiment, the SOS response is not induced (compare to A-D before 60 min). Whereas an subsequent induction of the SOS response can still be detected if the medium contained 1 µg/ml gentamicin **(F)**, it is further reduced at 10 µg/ml **(G)** and a complete block of the capacity of the cells to induce the SOS response is observed at 100 µg/ml gentamicin **(H)**. Consequently, Stx1 expression is completely abolished at 100 µg/ml gentamicin.

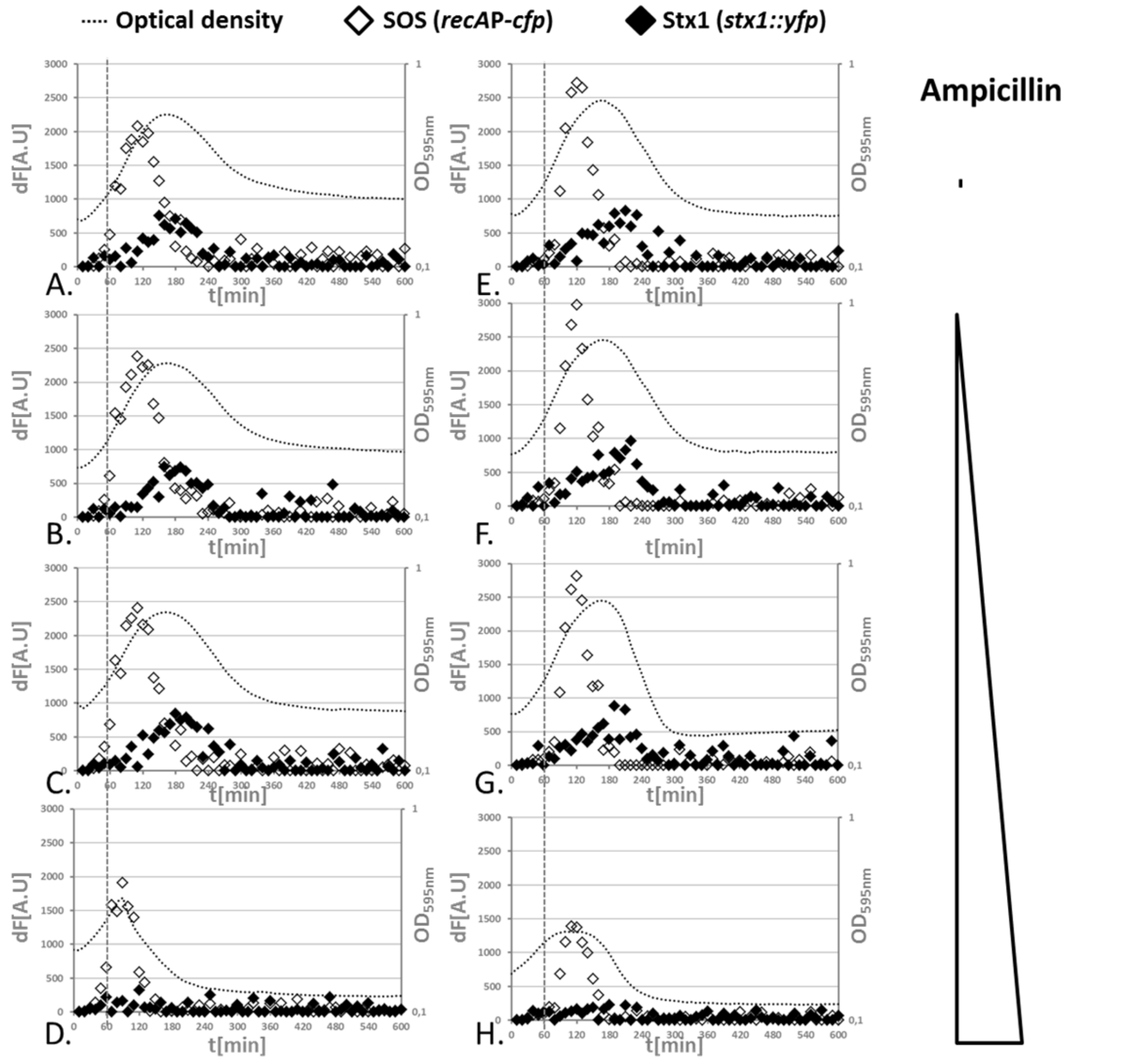

Supplementary Figure 6. Ampicillin does not block SOS response and Stx1 expression in EHEC O105:H7 EDL933 in a concentration dependent manner. Shown are growth curves (black dots) and SOS response (*recA*P-*cfp*, open diamonds) and Stx1 expression (*stx1::yfp*, black diamonds) as increase of fluorescence over time. **(A-D)** Ampicillin (grey dashed line) cannot block completely Stx1 expression after the SOS response was induced by diluting the cells in medium containing 0.1 µg/ml ciprofloxacin **(A).** Adding ampicillin to an f. c. of 1 µg/ml **(B)** has little effect on SOS response and Stx1 expression, as well as adding 10µg/ml ampicillin **(C).** At 100 µg/ml ampicillin, the SOS response is affected, but considerably less than for transcriptional and translational inhibitors and protein expression apparently continues, albeit at lower rates and Stx1 expression is reduced **(D)**. **(E-D)** Ampicillin cannot prevent SOS response and Stx1 expression induction by 0.1 µg/ml ciprofloxacin (grey dashed line). If the medium contains ampicillin from the beginning of the experiment, the SOS response is not induced (compare to A-D before 60 min). A subsequent induction of the SOS response can still be detected if the medium contained 1 µg/ml ampicillin **(F)**, at 10 µg/ml ampicillin **(G)** and at 100 µg/ml ampicillin **(H)**. Consequently, Stx1 expression is not completely abolished at 100 µg/ml ampicillin as well.

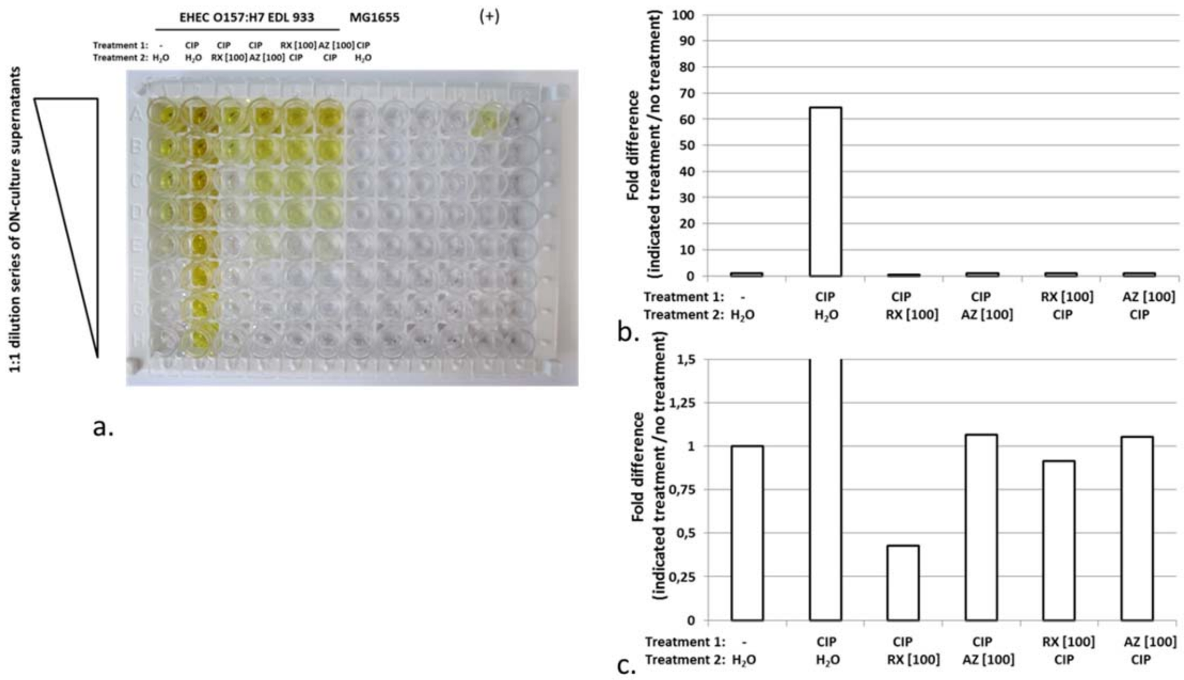

Supplementary Figure 7. Immunological detection of Stx1/ 2 in culture supernatants of EHEC O157:H7 EDL933. (a) Image of the ELISA performed with culture supernatants of EHEC O157:H7 EDL933 after the indicated treatment. *E. coli* K-12 MG1655 served as negative control, the positive control (+) was supplied together with the kit. The experiment was performed similar to the reporter gene assays, except that the bacteria were grown in glass tubes (for details see methods). **(b)** The graph shows the fold difference between the indicated treatment and the no treatment control. **(c)** The graph shows the same data as shown in (b), but to another scale. Rifaximine and azithromycin are keeping Stx1 /2 at or below the level of the no treatment control. CIP = ciprofloxacin (constant at 0.1 µg/ml), RX = rifaximine (c in µg/ml in brackets), AZ = azithromycin (c in µg/ml in brackets).

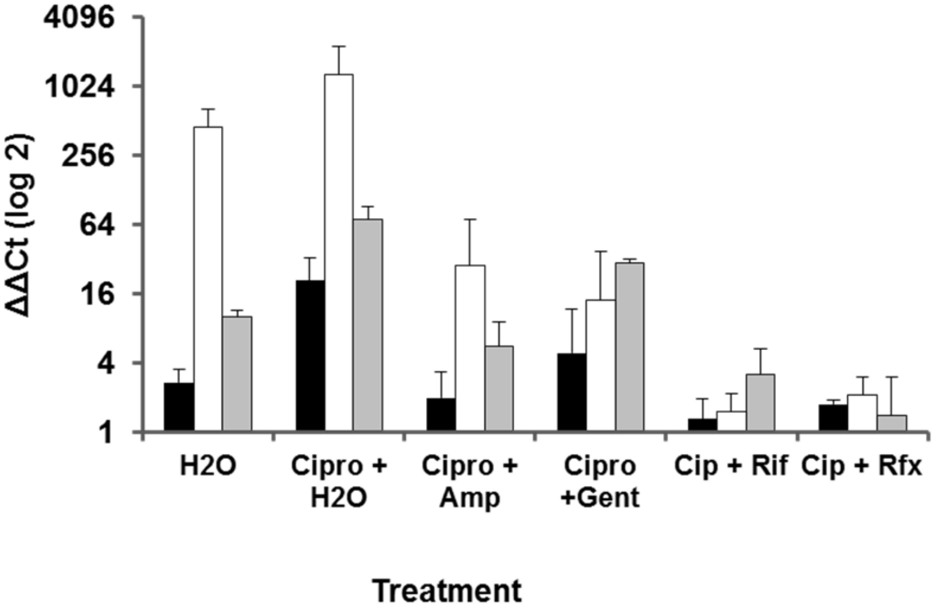

Supplementary Figure 8. Effect of antibiotic addition on transcript levels of *umu*D (SOS response), *stx1*A and *stx2*A in EHEC O107:H7 EDL933. Shown are the $\Delta\Delta C_T$ values obtained by comparing transcript levels of measured by RT-qPCR for *umu*D (black bars), *stx1*A (white bars) and *stx2*A (gray bars) relative to the housekeeping gene *uid*. RNA was isolated from cells grown in M9 medium supplemented with casamino acids without 2hr, with ciprofloxacin alone for 2 hr or with 0.1 μg/mL ciprofloxacin for 1 hr followed by growth in the presence of ampicillin (Amp., 100 μg /ml), gentamicin (Gent., 50 μg /ml), rifampicin (Rif., 100 μg /ml), or rifaximin (Rfx., 100 μg /ml) for an additional 1 hour, as indicated.

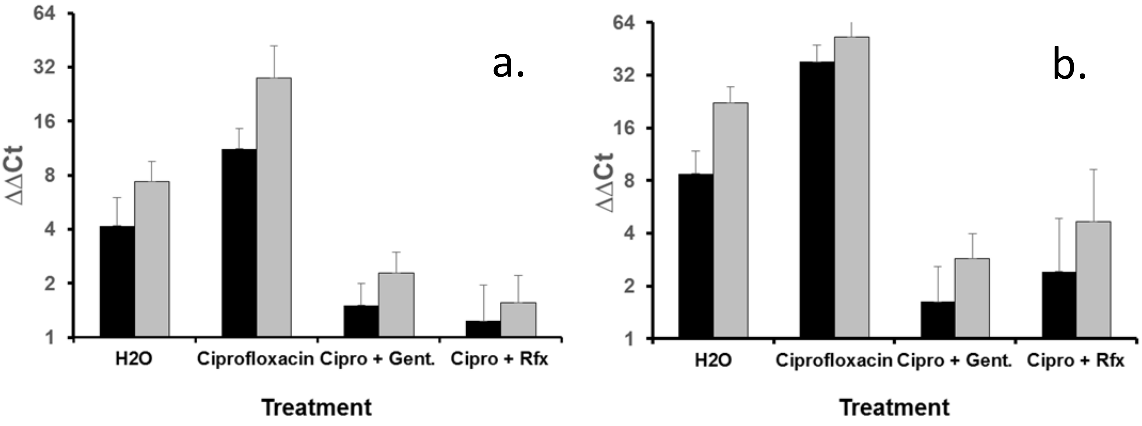

Supplementary Figure 9. Effect of antibiotic addition on transcript levels of *umu*D (SOS response), and *stx2* in EHEC HUSEC-1 (a) and HUSEC-41 (b). Shown are the $\Delta\Delta C_T$ values obtained by comparing transcript levels of measured by RT-qPCR for *umu*D (black bars), *stx1*A (white bars) and *stx2*A (gray bars) relative to the housekeeping gene *uidA*. RNA was isolated from cells grown in M9 medium supplemented with casamino acids without 2hr, with ciprofloxacin alone for 2 hours or with 0.1 μg/mL ciprofloxacin for 1 hour followed by growth in the presence of gentamicin (Gent., 50 μg /ml), or rifaximin (Rfx., 100 μg /ml) for an additional 1 hour, as indicated.

	**MIC [µg/ml]**
ciprofloxacin	0.08
ampicillin	5
rifaximine	12.5
azithromycin	12.5
gentamicin	10
tetracycline	1.25

A.

	0.1 µg/ml	1 µg/ml	10 µg/ml	100 µg/ml
ciprofloxacin	**1.25**	**12.5**	**125**	1250
ampicillin	0.02	**0.2**	**2**	**20**
rifaximine	0.008	**0.08**	**0.8**	**8**
azithromycin	0.008	**0.08**	**0.8**	**8**
gentamicin	0.01	**0.1**	**1**	**10**
tetracycline	**0.08**	**0.8**	**8**	80

B.

Supplementary Table 1. **(A)** MICs for the indicated antibiotic (for details see supplementary methods). **(B)** Concentrations of the indicated antibiotic expressed as fold-MIC (in bold: fold-MICs that were used in this study).

**Bacterial strain**	**Reference and/or construction**
EHEC O157:H7 EDL 933	Perna NT *et al* 2001
EHEC O157:H7 EDL 933 *Δstx1/2*	Gobert AT *et al* 2007
EHEC O157:H7 EDL 933 *Δstx1/2 stx1::yfp*	This study
E. coli K-12 MG1655	Blattner FR *et al* 1997
E. coli K-12 MG1655 recAP-*cfp-aph*	This study
HUSEC 1	Mellmann A *et al* 2008
HUSEC 41	Mellmann A *et al* 2008

Supplementary Table 2: Bacterial strains used in this study.

Primer	Sequence (5´-> 3´)
MBP 5	GGATGAATGGCAGAAATTCG
MBP 185	GTCACGCAGCGTCAGTGAA
MBP 206	GCTGAACTTGTGGCCGTTTA
MBP 224	GGATGTCAAGAATATAGTTATCGTATGGTGCTCAAGGAGTATTGTGTAATATGGTGTCTATCACTAAAGATC
MBP 225	ATTTAACATTTATGAATCTCCGCCTGCTATTTTCACTGAGCTATTCTAAGAGGAAACAGCTATGACCATG
MBP 228	GTGGTTACATTGCCACGCTG
MBP 254	AGAAAACGGCCCACCGTATG
MBP 262	CAACAGAACATATTGACTATCCGGTATTACCCGGCATGACAGGAGTAAAAATGGTGTCTATCACTAAAGATC
MBP 263	TAACTGAAAAGTGGGAATAAGATAAGTTTTCTTGACTGGGAAGTAAAATAAGGAAACAGCTATGACCATG
MBP 265	GCGGTGAGTGAACCCGTCGT
MBP 266	CGGCGTTGGTAAACAACAAAG
uidA_F	CAGTCTGGATCGCGAAAACTG
uidA_R	ACCAGACGTTGCCCCACATAATT
umuD_F	CCCGACGGTACAGCTTATTC
umuD_R	GTGGATCACCACACCAAAGA
stx1_F	GTGGCATTAATACTGAATTGTCATCA
stx1_R	GCGTAATCCCACGGACTCTTC
stx2a_F	GATGTTTATGGCGGTTTTATTTGC
stx2a_R	TGGAAAACTCAATTTTACCTTTAGCA

Supplementary Table 3: Primers used in this study.

Supplementary Literature

1. Datsenko, K. A. & Wanner, B. L. One-step inactivation of chromosomal genes in *Escherichia coli* K-12 using PCR products. *Proc. Natl. Acad. Sci. U. S. A.* **97**, 6640-6645 (2000).

2. Gobert, A. P. *et al*. Shiga toxin produced by enterohemorrhagic *Escherichia coli* inhibits PI3K/NF-kappaB signaling pathway in globotriaosylceramide-3-negative human intestinal epithelial cells. *J. Immunol.* **178**, 8168-8174 (2007).

3. Berger, M. *et al*. Genes on a Wire: The Nucleoid-Associated Protein HU Insulates Transcription Units in *Escherichia coli*. *Sci. Rep.* **6**, 31512 (2016).

4. Wang, R. F. & Kushner, S. R. Construction of versatile low-copy-number vectors for cloning, sequencing and gene expression in *Escherichia coli*. *Gene* **100**, 195-199 (1991).

5. Friedman, N., Vardi, S., Ronen, M., Alon, U. & Stavans, J. Precise temporal modulation in the response of the SOS DNA repair network in individual bacteria. *PLoS Biol.* **3**, e238 (2005).
